# Permanent First Mandibular Molar: Loss Prevalence and Pattern among Saudis in Al-Ahsa

**DOI:** 10.1055/s-0042-1757904

**Published:** 2022-12-10

**Authors:** Hatim Mohammed Almahdi, Zuhair Alabdrabulridha, Jawad AlAbbas, Ali Albin Saad, Ismail Alarka, Sajjad Alghatm, Hadi Alqasem

**Affiliations:** 1 Department of Oral and Maxillofacial Surgery and Diagnostic Sciences, College of Dentistry, King Faisal University, Al-Ahsa, Saudi Arabia; 2 Dentist in King Faisal University Dental Complex, Al-Ahsa, Saudi Arabia

**Keywords:** caries, permanent first mandibular molar, Saudi Arabia, tooth extraction

## Abstract

**Objectives** The permanent first mandibular molar (PFMM) is the first tooth to erupt, usually at the age of 6 years. It is the most effective oral masticatory unit due to its wide occlusal surface and its role in favorable occlusion. This study describes the prevalence of PFMM loss and the reasons for extraction in a group of King Faisal Dental Complex Clinics (KFUDC) Saudi attendees. In addition, to report postextraction complications, consider the gender, age, and the time elapsed from the moment of the extraction.

**Materials and Methods** A cross-sectional study was performed in January to April 2020, focusing on the loss of PFMM among a group of Saudis attending KFUDC in Al Hofuf, Saudi Arabia. A total of 417 participants were recruited. The inclusion criteria were adults aged ≥ 18 years. The principal investigator performed all the necessary measures for calibration in the forms of training on clinical examination and interviews.

**Statistical Analysis** Data were analyzed using the Statistical Package for the Social Science, version 25 (SPSS Inc., Illinois, United States).

**Result** Four hundred seventeen attendees participated in the present study. Majority were males 73.9% (308), and 26.1% (109) females. A third, 30% (125), reported missing PFMM; the overwhelming majority reported the reason for extraction as caries 93.6% (117).

On clinical examination, 80.8% (101) had complications; drifting of adjacent teeth was the most common complication, 57.6% (72), followed by supraeruption in 23.2% (29).

More males than females reported missing PFMM (22.8 and 7.2%, respectively), and those ≥ 25 years reported more missing PFMM than younger (17.5 and 12.5%, *p* ≤ 0.000). Those confirmed with systemic diseases reported more missing PFMM than their counterpart (23.3 and 6.7%, *p* ≤ 0.01).

Those with good oral hygiene reported less missing PFMM than their poor oral hygiene counterparts (27.8 and 34%). Those who had good knowledge about the complications of early extraction of PFMM stated less missing PFMM than their counterparts (15.6 and 14.4%, *p* ≤ 0.01).

**Conclusion** This study indicated that PFMM were the most common extracted tooth. Caries is the principal reason for tooth extraction among the studied population, followed by periodontal diseases. Emphasis on preventing dental caries is essential to maintain a socially and economically productive life and reduce the burden of oral disease.

## Introduction

The permanent first mandibular molar (PFMM) is the first tooth to erupt, usually at the age of 6 years. It is the most effective oral masticatory unit because of its wide occlusal surface, and it plays a fundamental role in favorable occlusion. Thus, they are considered essential permanent tooth because of its several roles in developing and maintaining occlusion. 1

The PFMM's position posterior to the second deciduous molar, and the eruption pattern, without any predecessors, make it the foundation of the dental arches. It also significantly coordinates horizontal, anterior-posterior, and transversal growth of both jaws, facial growth, and facial height. 2 Furthermore, it is vital for other permanent teeth erupt in a favorable position and proper occlusion.

The PFMM is the most caries-prone tooth as it is exposed to the oral environment for a more extended period than any other permanent tooth. In addition, it tends to have deeper pits and fissures that trap food, resulting in dental caries and tooth loss. 3 4

Several studies have described that loss of permanent molars produces adverse effects on occlusion, such as tilting of neighboring teeth, supraeruption of the teeth in the opposite arch, unilateral chewing, and shift in the dental midline and dental malocclusion. Moreover, it leads to decreased masticatory force. 5 6

Worldwide, dental caries and periodontal diseases are the main reasons for extraction. 7 8 9 10 The first and third mandibular molars were the most frequently extracted posterior teeth, and the prevalence of PFMM loss was 10.9 to 22.2%. 10 11 12

This study describes the prevalence of PFMM loss and the reasons for extraction in a group of Saudi attendees of King Faisal Dental Complex Clinics (KFUDC). Moreover, to report postextraction complications, considering the patients' gender, age, and the time elapsed since extraction.

## Material and Methods

A cross-sectional study was conducted from January to April 2020, focusing on PFMM loss among a group of Saudis attending KFUDC in Al Hofuf, Eastern Province, Saudi Arabia. KFUDC is a major dental center serving King Faisal University with more than 90,000 employees and students. 13 As of 2021 census, the city of Al Hofuf had 858,395 inhabitants. 14

A total of 417 participants were recruited from KFUDC attendees. The inclusion criteria were adults aged ≥ 18 years; the exclusion criteria were uncommunicative with various mental disabilities. The investigators completed clinical examinations using a dental examination set. Data from the clinical examination were recorded on a form and supplemented with interview answers. The clinical examination and interview techniques were calibrated using all the necessary measures during the training sessions.

An interview was completed in KFUDC by trained data collectors. They provided standardized instructions about the study's purpose and the interview process to the participants. The interview was constructed in English and administered in Arabic. A pilot study testing the translation's accuracy and the questions' clarity was conducted before the study's administration among selected attendees. Some minor adjustments to the survey instrument were performed before administering the survey.

Data collection sheet consisted of two parts: the questionnaire and the clinical examination. The questionnaire contained three domains. The first was about the patients' demographics (age, gender, and occupation). The second comprised questions to evaluate the patients' oral health status. The third consisted of questions about the cause of PFMM loss and knowledge about complications. The clinical examinations were done using a dental examination set to check the presence of PFMMs and other teeth, supraeruption of opposing teeth, and drifting of adjacent teeth.

### Data Analysis

Data were analyzed using the Statistical Package for Social Science, version 25 (SPSS Inc., Illinois, United States). Descriptive analyses using frequencies and percentages. Bivariate relationships between the dependent and independent variables were analyzed using cross-tabulation and the chi-squared test.

## Results

Four hundred seventeen attendees participated in the present study. Of them, 308 (73.9%) were males, and 109 (26.1%) were females; 229 patients (54.9%) were younger than 25 years old, and 188 patients (45.1%) were 25 years of age or older. Only 89 patients (21.3%) were unemployed. Most of the attendees were healthy (351 patients; 83.2%) ( Table 1 ).

**Table 1:** Frequencies ( *n* ) and percentages (%) of the sociodemographic characteristics of participants

Variable	% ( *n* )
Gender	
Female	26.1 (109)
Male	73.9 (308)
Total	100 (417)
Employment	
Employed	78.7 (328)
Not employed	21.3 (89)
Total	100 (417)
Age group	
< 25 years	54.9 (229)
≥ 25 years	45.1 (188)
Total	100 (417)

Regarding oral health parameters, most of the participants (207 patients; 64.7%) reported visiting dental clinics only when needed, whereas 97 (23.3%) regularly visited the dentist, and 50 (12%) reported that the study visit was their first-ever visit. Most participants used a toothbrush (404 patients, 96.9%), and the rest used *miswak* ( *stick from a plant used as a toothbrush* ). Regarding brushing frequency, 72 patients (41.2%) brushed once a day, 187 (44.8%) brushed twice a day, and 30 (7.2%) never brushed their teeth. In terms of oral health aids used in addition to brushing, 241 patients (57.8%) used no aids, 89 (21.3%) used floss, and 87 (20.9%) used mouthwash. Additionally, 99 patients (23.7%) used tobacco.

About one-third of the patients (125 patients; 30%) reported missing PFMMs, the overwhelming majority of whom (117 patients; 93.6%) reported caries as the reason for extraction. Other reasons included periodontal diseases, trauma, and orthodontic treatment. Regarding time elapsed since extraction among those reporting missing PFMMs, 83 patients (66.4% of those with missing PFMMs) reported periods of less than 5 years, whereas 42 patients (33.6% of those with missing PFMMs) reported periods of 5 years or more ( Table 2 ). Based on clinical examination, 101 (80.8% of patients with missing PFMMs) had complications. Drifting of adjacent teeth was the most common complication, seen in 72 patients (57.6% of patients missing PFMMs), followed by supraeruption, seen in 29 patients (23.2% of patients missing PFMMs). Regarding knowledge of complications of missing PFMMs, 357 patients (85.6%) had poor knowledge, and 14.4% (60) had good knowledge.

**Table 2:** Frequencies ( *n* ) and percentages (%) of variables related to loss of PFMM among the participants

Variable	% ( *n* )
Loss of PFMM	
No	70 (292)
Yes	30 (125)
Total	100 (417)
Reasons for loss of PFMM	
Caries	93.6 (117)
Periodontal diseases	2.4 (3)
Others	4 (5)
Total	100 (125)
Oral Hygiene habits	
Poor	34.5 (144)
Good	65.5 (273)
Total	100 (417)
Knowledge about complications of loss of PFMM	
Poor	74.6 (311)
Good	25.4 (106)
Total	100 (417)
Presence of complications	
No	75.7 (316)
Yes	24.3 (101)
Total	100 (417)

Abbreviation: PFMM, permanent first mandibular molar.

More males (22.8%) than females (7.2%) reported missing PFMMs, and patients aged 25 years or older were more likely to report missing PFMMs (17.5%) than younger patients (12.5%; *p* ≤ 0.000; Table 3 ). Those with good oral hygiene were less likely to report missing PFMMs (27.8%) than their counterparts with poor oral hygiene (34%). Patients who had good knowledge about the complications of early PFMM removal were less likely to report missing PFMMs (14.4%) than their counterparts with poor knowledge (15.6%, *p* ≤ 0.01; Table 3 ).

**Table 3:** Frequencies ( *n* ) and percentages (%) by to loss of PFMM among the participants by gender, age, oral hygiene health, and knowledge of complications

Variable	% ( *n* )
Gender	
Female	7.2 (30)
Male	22.8 (95)
Total	30 (125)
Age group	
< 25 years	12.5 (52)
≥ 25 years	17.5 (73) a
Total	30 (125)
Oral hygiene health	
Poor	11.8 (49)
Good	18.2 (76)
Total	30 (125)
Knowledge about complications of loss of PFMM	
Poor	15.6 (65) a
Good	14.4 (60)
Total	30 (125)

Abbreviation: PFMM, permanent first mandibular molar.

a *p* ≤ 0.000.

## Discussion

Many studies have been conducted worldwide to investigate tooth loss. This study was focused on assessing the prevalence of PFMM extraction, the reasons for extraction, and its most common complications, considering patients' gender, age, and other factors in a group of Saudi KFUDC attendees.

This study confirmed the rate of PFMM loss to be 30% among the participants, similar to other studies from Saudi Arabia, the United Arab Emirates, and Iran. 10 15 16 17 18 A study from another region in Saudi Arabia confirmed that PFMMs are the most commonly extracted teeth but in a lower percentage of individuals than reflected in our study. 19

The current study demonstrated that missing PFMMs are more common among males than females, but the difference was statistically insignificant. This result agrees with other studies. 16 19 20 Similar percentages were found in males and females in Jizan region but not with a study in Turkey, where females were more likely to report missing PFMMs than males. 21 22 This disparity might be attributed to the fact that females visit the dentist more than males and care more about aesthetics and oral health than males do. 23

Our findings showed that caries is the most common cause of teeth extraction, followed by periodontal diseases. These problems are attributed to high dietary intake of refined sugar, inadequate oral hygiene, and lack of awareness regarding timely dental check-ups in these populations ( Table 2 ). These findings were consistent with those of other studies conducted in Saudi Arabia. 16 19 22 24

The consequences of PFMM removal include drifting and tipping of adjacent teeth and overeruption of opposing teeth, which are the most common complications, as well as horizontal and vertical bone resorption. In this study, the overwhelming majority of patients with extracted PFMMs had developed complications, with drifting of adjacent teeth being most common, followed by supraeruption. 25 26

This study confirmed that those with good oral hygiene and good knowledge about the complications of early PFMM extraction were less likely to report missing PFMMs than their counterparts with poor hygiene and awareness. This is similar to studies comparing oral hygiene, knowledge, and tooth loss. 27 28 Retention of teeth is reflected in the oral-health-related quality of life among patients from other studies. 29 30

Based on the progressive and rapid changes in the country's development in recent years, enormous changes have been generated at educational, cultural, and social levels. Patients are expected to be increasingly aware of oral health and the importance of preserving teeth for a better quality of life. Additionally, social media is involved in closing the knowledge gap between dentists and patients. However, they were highly similar when comparing tooth loss patterns between patients in the current study and those in previous studies conducted in Saudi Arabia. 31

The present study has some limitations that should be considered before any possible benchmarking with similar studies. Few similar studies have been conducted in the Kingdom of Saudi Arabia and included different populations or aspects of comparison. The present study include individuals who sought dental treatment at KFUDC; therefore, the sample could not be considered representative of the population, and participants were subjected to selection and information (recall) bias. Despite these limitations, it describes the current situation of oral health in the study area.

## Conclusion

This study indicated that PFMMs were the most common set of teeth to be extracted. Caries is the principal reason for tooth extraction in the studied population, followed by periodontal diseases. Emphasis on preventing dental caries is essential to maintain a socially and economically productive life and reduce the burden of oral disease.
